# Young patients’ self-reported fear compared to professionals’ assessments during invasive and non-invasive dental visits: a prospective, longitudinal study

**DOI:** 10.1007/s40368-021-00685-4

**Published:** 2022-01-12

**Authors:** L. Krekmanova, M. Sotirianou, N. Sabel

**Affiliations:** 1 Department of Pediatric Dentistry, Institute of Odontology, Sahlgrenska Academy, University of Gothenburg, Gothenburg, Sweden; 2 Clinic of Pedodontics, Public Dental Service, Region Västra Götaland, Gothenburg, Sweden; 3 Public Dental Service, Region Västra Götaland, Gothenburg, Sweden

**Keywords:** Child, Adolescent, Dental fear, Self-report, Proxy report

## Abstract

**Purpose:**

The main purpose was to study young patients’ self-reports on dental fear over a 5-year period, prospectively. Also, to compare these to professionals’ proxy reports for dental fear during invasive and non-invasive dental visits. The research question was, to what extent the self-reports and dental professionals’ proxy reports are congruent, and if there were patient age-dependent differences.

**Methods:**

3134 patients from 11 public dental clinics, representing urban and rural areas, were invited. Four age cohorts were formed: 3, 7, 11, and 15 years of age and followed between the years 2008 and 2012. Dental examinations (non-invasive) and restorative treatments + extractions (invasive) were registered. During the treatments, self-reports regarding fear and professional proxy reports were registered: Not afraid at all = 0, little nervous = 1, quite afraid = 2, very scared = 3, terrified = 4.

**Results:**

2363 patients completed the cohort periods (51% girls and 49% boys). In all, 9708 dental examinations, restorations and extractions were performed. The fear prevalence increased with the invasiveness of the dental procedure; 7–56%. For dental examinations and restorations, fear declined with ascending age. The highest fear prevalence was reported for dental extractions. Younger children reported fear more frequently than older children, *p* < 0.001. Frequent inconsistencies between self-reports and proxy reports were observed among the younger children (16%) compared to the older children (8%), *p* < 0.001.

**Conclusion:**

Non-congruence was observed for self-reports and proxy reports regarding all age cohorts.

## Introduction

Despite experiencing fear, children and adolescents often cooperate during dental procedures, such as examinations, restorations, or extractions (Ghanei et al. 2018; Krekmanova et al. 2009; Krekmanova and Robertson 2020). However, some children and adolescents have difficulty to interrupt and stop an ongoing dental treatment. Dental fear and anxiety (DFA) may be triggered by temperament, upbringing, and patient–dentist-dependence, as well as insensitive dentists (Klingberg and Broberg 1998), (Wondimu and Dahllof 2005). In addition, the perceived DFA or the perception of being compelled to cooperate may magnify the negative experience. Also, negative expectations are known as powerful modulators for future experiences (Williams et al. 2015). Therefore, dental professionals’ sensitivity to the young patient’s subtle DFA reaction is critical (Krekmanova et al. 2009). In contrast, behavior management problems (BMPs) are detectable by the unwillingness to cooperate (Klingberg 1995).

The Convention on the Rights of the Child highlights that all children should be given the possibility to express their own needs and have access to the best available medical methods (UNICEF). Consequently, children need to be given an opportunity to deal with and learn to feel safe during dental procedures. They should be offered reasonable chances to deal with challenging circumstances and to develop an understanding for the dental care session. Patient cooperation in the long run may positively reinforce the relation with the dental team. Based on the above, dental professionals should identify DFA while treating young patients (Klingberg 1995; Krekmanova et al. 2009; Krekmanova et al. 2009; Krekmanova and Robertson 2020).

Children’s dental fear is best communicated through self-reports, which constitutes the golden standard (Measelle et al. 2005). On the other hand, proxy reports reinforce or compensate for the individual’s insufficient maturity or ability to verbally communicate the own needs. The medical literature holds considerable knowledge on the congruence between children's self-reports and proxy assessments on pain (Mack et al. 2020). Often, these studies focus on medically ill children and highlight the inconsistency between their experiences and the proxy assessments (Pinheiro et al. 2018). The dental literature holds some information on the congruence between young patients’ fear and the dental professionals’ estimation during dental sessions. A poor agreement between self- and proxy reports is found in the literature (AlGharebi et al. 2021; Klein et al. 2015; Klingberg and Broberg 2007; Krikken et al. 2013; Luoto et al. 2010; Morgan 2015; Patel et al. 2015; Tollili et al. 2020). However, there is a knowledge gap regarding longitudinal prospective studies performed on a larger scale. Therefore, the aim was to study young patients’ self-reports on fear in comparison to professionals’ proxy reports during invasive and non-invasive dental visits, over a five-year period, prospectively.

## Methods

### Study design and patients

This study was approved by the Swedish Public Dental Service in Region Västra Götaland and Region Örebro County, and the National Ethical Review Board. The survey is a 5-year cohort study of an accelerated, longitudinal design.

3134 children and adolescents aged 3–15 years were eligible and invited to participate. The individuals were geographically seen at 11 Public Dental Clinics, selected to represent a young population from urban and rural areas. All parents gave written consent to their child’s participation. Also, each child gave assent through a written consent if ≥ 12 years old, or together with a parent if ≤ 12 years old.

In 2008 at baseline, four age cohorts were formed: 1 = 3 ≥ 7 years old, 2 = 7 ≥ 11 years old, 3 = 11 ≥ 15 years old, and 4 = 15 ≥ 19 years old. E.g., those children who turned 11 years old are only included in Group 3. Each child was regularly followed up till 2012 through an individually determined dental care need and oral health revision. Consequently, the children in Group 3 were not consistent with those children in Group 3 5 years later; an accelerated, longitudinal design.

The eligible licensed general dentists were working full or part time at the 11 participating clinics. Their professional experience varied, which reflected the actual circumstances. However, they were calibrated beforehand regarding the outcome measures.

### Clinical registrations

For this study’s analysis, dental examinations were defined as *non-invasive* treatment, while restorative dental treatments and/or extractions were defined as *invasive* treatment. During each dental session, the operating professional registered the performed treatments. If any invasive treatment was included during the dental session, the session was classified as invasive treatment.

### Self-reported DFA

Each patient was given the possibility to assess and report a possibly perceived fear, subsequent to each dental session, by answering the question: How did you feel today? Not afraid at all = 0, little nervous = 1, quite afraid = 2, very scared = 3, terrified = 4.

In cases where the youngest children had difficulty with self-reporting, parental help was used.

### Proxy-reported DFA

Each concerned dental professional gave a proxy report on the patient’s fear using the DFA graded scale, subsequent to each dental session: Did the patient experience DFA? Not afraid at all = 0, little nervous = 1, quite afraid = 2, very scared = 3, terrified = 4.

The dental professionals were calibrated on the DFA outcome measures through meetings before the study start.

### Statistical methods

Statistical data described patients, gender, and dental examinations (non-invasive dental treatments), as well as restorations and extractions (invasive treatment).

The self-reported DFA was dichotomized into new variables; Not afraid at all (0) = 0, and Afraid (1–4) = 1. The dichotomization of DFA scores in the current study was performed to clearly separate the children who were not afraid, from the children who were somewhat nervous or more; DFA ≥ 1.

The self-reported DFA prevalence (%) was calculated for each age cohort, respectively. The age cohorts were also dichotomized into new variables: Younger children (Cohorts 1 + 2), and Older children (Cohorts 3 + 4). Chi-square tests were applied on dichotomized variables to analyze for possible significant DFA differences.

The congruencies and discrepancies between patients’ self-reported DFA and professional proxy reports were calculated. The corresponding congruencies were calculated also when DFA was dichotomized. Thereby, DFA scores 1–4 were grouped together, versus DFA = 0. The patient’s positive DFA (1–4) and the corresponding positive proxy report (1–4) were considered congruent. Also, the patient’s negative DFA (0) self-report and the corresponding negative proxy report (0) were considered congruent.

The inconsistency between the DFA reports (positive self-report/negative proxy report, or vice versa) conveyed a discrepancy.

The sensitivity and specificity, as well as the positive predictive value (PPV) and the negative predictive value (NPV), were calculated for the self-reported and professional proxy reports of DFA.

IBM SPSS Statistics for Windows, Version 26.0 (IBM Corp., NY, USA), was used for the statistical analyses, and *p* values below 0.05 represented statistical significance.

## Results

Out of the 3134 eligible registered children at baseline, 2363 completed the cohort periods: 1: (3–7 years) *n* = 695 children, 2: (7–11 years) *n* = 642 children, 3: (11–15 years) *n* = 574 children, and 4: (15–19 years) *n* = 452 children, in total 1215 girls (51%) and 1148 boys (49%).

During the study period, 9708 dental sessions with both self-reported and professional proxy reports of DFA were registered; 8070 sessions of dental examinations, and 1202 sessions of restorations, as well as 436 sessions of extractions, were completed (Table 1). The self-reported DFA prevalence (%) ranged with the invasiveness of the dental procedure by 7–57%. The frequencies and prevalence for each age cohort and dental treatment are presented in Table 1.

**Table 1:** The age-cohorts’ corresponding treatment sessions (n) and DFA-prevalence (%) regarding dental checkup, tooth restoration, and tooth extraction respectively. Younger children (cohort 1+2) compared with older children (cohort 3+4) show higher prevalence of DFA for dental checkup and for tooth restoration

Cohort	1	2	3	4	Total
Age	3–7 years	7–11 years	11–15 years	15–19 years	
Treatment sessions (*n*)					
Dental examinations^a^	2250	2250	1968	1602	8070
Dental restorations^b^	167	397	312	326	1202
Dental extractions^b^	35	164	179	46	424

Prevalence DFA (%)					Mean	*p* value*
Dental examinations^a^	19	20	12	7	15	< 0.001
Dental restorations^b^	35	38	35	21	32	< 0.001
Dental extractions^b^	46	52	57	44	53	n.s

*n.s.* not significant

*Chi-square tests performed between Cohorts 1 + 2 vs Cohorts 3 + 4

^a^Non-invasive treatments

^b^Invasive treatments

For both dental examinations and restorations, the self-reported DFA declined with age. The DFA prevalence (%) was highest for the extractions and varied only slightly between the age cohorts (Table 1). The gender distribution at baseline was reflected in both the non-invasive as well as the invasive treatments (Table 2).

**Table 2:** Each age cohort’s gender distribution, non-invasive and invasive treatment sessions (*n*)

Cohort	1			2			3			4		
Treatment (*n*)	Girl	Boy	Total	Girl	Boy	Total	Girl	Boy	Total	Girl	Boy	Total
Non-invasive	1149	1101	2250	1085	1165	2250	1057	911	1968	843	756	1602
Invasive	116	89	205	288	263	551	252	228	480	220	151	371

The chi-square analysis for the dichotomized DFA showed a significant statistical difference since younger children reported fear more frequently, compared to older children, *p* < 0.001 (Table 1). The corresponding analysis for the dental extractions showed a non-significant difference, *p* > 0.05 (Table 1).

Considering the dental treatments in general, a more frequent inconsistency between DFA reports was observed regarding younger children (16%), compared to older children (8%), *p* < 0.001. This was also true for non-invasive sessions with younger children (449 sessions; 12%), compared to older children (231 sessions; 7%), (Table 3). Considering DFA for the invasive treatments and younger children’s 106 sessions (15.7%), compared to 84 sessions (10.8%) of older children, the statistical difference was also significant, *p* < 0.05 (Table 4).

**Table 3:** Non-invasive treatment. DFA reports (n) and percentages (%) presented for each cohort. Patient’s report/professional’s report. Coherence between patient’s self-report and professional’s proxy report when DFA dichotomized. Pos: DFA score: 1–4; neg: DFA score: 0 (0 = no fear, 1–4 = dental fear). pos/pos = patient and professional consensus of opinion on patient’s DFA > 0 and neg/neg = patient and professional consensus of opinion reporting the patient’s DFA = 0

DFA reports					
Cohort	1	2	3	4	*p* value
Age	3–7 years *n* (%)	7–11 years *n* (%)	11–15 years *n* (%)	15–19 years *n* (%)	
pos/pos	335 (20)	304 (14)	150 (8)	65 (4)	
pos/neg	061 (4)	134 (6)	86 (5)	52 (4)	
neg/pos	139 (9)	115 (6)	74 (4)	19 (1)	
neg/neg	1103 (67)	1552 (74)	1515 (83)	1348 (91)	
Total	1638 (100)	2105 (100)	1825 (100)	1484 (100)	
Inconsistency^a^	200 (12)	249 (12)	160 (9)	71 (5)	< 0.001*
Coherence^b^	1438 (88)	1856 (88)	1665 (91)	1413 (95)	

*pos/neg* and *neg/pos* inconsistency between patient’s DFA self-report and professional’s proxy report

*Chi-square tests performed of inconsistency between Cohorts 1 + 2 vs Cohorts 3 + 4

^a^pos/neg and neg/pos (patient and professional inconsistency on patient’s DFA)

^b^pos/pos and neg/neg (patient and professional consensus of opinion on patient’s DFA)

**Table 4:** Invasive treatment

DFA reports					
Cohort	1	2	3	4	*p* value
Age	3–7 years *n* (%)	7–11 years *n* (%)	11–15 years *n* (%)	15–19 yrs *n* (%)	
pos/pos	60(36)	188(37)	170 (39)	75 (22)	
pos/neg	008 (5)	32 (6)	21 (5)	10 (3)	
neg/pos	19 (11)	47 (9)	29 (6)	24 (7)	
neg/neg	80 (48)	242 (48)	218 (50)	228 (68)	
Total	167 (100)	509 (100)	438 (100)	337 (100)	
Inconsistency^a^	27 (16)	79 (16)	50 (11)	34 (10)	< 0.05*
Coherence^b^	140 (84)	430 (84)	388 (89)	303 (90)	

DFA reports (*n*) and percentages (%) presented for each cohort. Patient’s report/professional’s report. Coherence between patient´s self-report and professional’s proxy report when DFA dichotomized. Pos: DFA-score: 1–4; neg: DFA-score: 0 (0 = no fear, 1–4 = dental fear)

*pos/pos* patient and professional consensus of opinion on patient’s DFA > 0 and *neg/neg* patient and professional consensus of opinion reporting the patient’s DFA = 0

pos/neg and neg/pos = inconsistency between patient’s DFA self-report and professional’s proxy report

^a^pos/neg and neg/pos (patient and professional inconsistency on patient’s DFA)

^b^pos/pos and neg/neg (patient and professional consensus of opinion on patient’s DFA)

*Chi-square tests performed on inconsistency; Cohorts 1 + 2 vs Cohorts 3 + 4

As seen in Table 5, sensitivity drops with increased age, while specificity rises with age.

**Table 5:** Non-invasive treatment and invasive treatment; sensitivity, specificity, *PPV* positive predictive values, and *NPV* negative predictive values for each cohort

Probability: non-invasive/invasive treatment				
Cohort	1	2	3	4
Age	3–7 years	7–11 years	11–15 years	15–19 years
Sensitivity	0.85/0.88	0.69/0.85	0.64/0.89	0.56/0.88
Specificity	0.89/0.81	0.93/0.84	0.95/0.88	0.99/0.90
PPV	0.71/0.76	0.73/0.80	0.67/0.85	0.77/0.76
NPV	0.95/0.91	0.92/0.88	0.95/0.91	0.96/0.96

The positive predictive values (PPV) and the negative predictive values (NPV) are presented in Table 5, the largest PPV data for invasive treatments; 0.85 (age cohort 11–15 years).

### DFA-fluctuations regarding the sexes

22.8% of the girls reported fear compared to 17.7% of the boys for all dental procedures, *p* < 0.001. The girls reported significantly more DFA regarding sessions which included dental restorations and examinations; *χ*^2^ *p* < 0.001. For sessions including extractions, no statistically significant differences were observed regarding the sexes’ DFA reports; *χ*^2^, *p* > 0.05. At sessions including extractions, 45% of the boys and 55% of the girls reported DFA (mean 53%).

The DFA analyses regarding the non-invasive and invasive dental procedures showed that 11% of the girls were mis-rated in comparison to 9% of the boys, *p* < 0.014. Regarding invasive treatment, 12.5% of the girls were mis-rated in comparison to 12% of the boys, *p* > 0.70. More girls (10%) than boys (8%) were mis-rated when it comes to non-invasive treatments, *p* < 0.014.

## Discussion

The main results showed the young patients’ self-reported DFA increased with invasive dental procedures. Furthermore, children < 11 years of age more frequently reported DFA during dental examinations and restorations than older children. The results also revealed that among 11–19-year-olds, 7–12% experienced fear when undergoing regular dental examinations. Regarding extractions, 41–56% of all 3–19-year-olds rated themselves as experiencing some degree of fear, which is considered noteworthy. The findings are in congruence with previous studies, indicating that preventive measures to minimize young patients’ DFA have not yet reached the desired outcome (Cianetti et al. 2017; Ghanei et al. 2018; Krekmanova and Robertson 2020).

DFA is a complex phenomenon with various intrinsic and extrinsic influencing factors that all are individually interpreted by the patient. Numerous instruments have been developed and are used in research settings to measure DFA’s various fear aspects (Porritt et al. 2013; Yon et al. 2020).

A frequently used tool in research is the Children's Fear Survey Schedule-Dental Subscale (CFSS-DS) (Klingberg 1994). However, a drawback for this instrument is its time-consuming nature, as this tool consists of 15 questions. It also requires the younger patient to be literate. In comparison, this current study design used two questions in the DFA self-report and proxy report.

Berggren and Meynert 1984, emphasized that DFA developed in adulthood is often triggered by traumatically experienced dental treatments during childhood (Berggren and Meynert 1984), showed that pre-schoolers treated for carious lesions are at a higher risk for developing DFA by 10 years of age (Raadal et al. 2002). Recurrent painful procedures and lack of control, as well as insensitive dentists, are often the triggers (Krekmanova et al. 2009). Today, up to 37% of the adult population still report DFA, apart from 5% reporting dental phobia for the same reasons (Svensson et al. 2016). Dental injection is found to be the highest ranked DFA-trigger among children and adolescents (Vanhee et al. 2020). Furthermore, some dentists consider themselves to be challenged by children < 10 years of age, having to meet their fear and uncooperativeness when using local anesthesia (Ronneberg et al. 2015).

Children with behavior management problems (BMP) frequently score high on the CFSS-DS (Wogelius et al. 2003). Therefore, dental care offered to children and adolescents requires a high level of expertise. Positive dental treatment experiences may lead to latent inhibition, i.e., a higher resistance for developing DFA (van Waaijen et al. 2001).

A notable result was the inconsistency between the patients’ DFA reports and the DFA proxy assessments as every fourth child between the ages of 3–11 years was mis-rated during regular dental examinations. The youngest of the age group could be considered especially vulnerable due to insufficient maturity and understanding of the own participation in the dental situation. This in itself could negatively affect the treatment outcome. No comparable data exist to confirm these findings. On the other hand, the inconsistency between the DFA self-reports and proxy reports may have mirrored the dental professional’s sensitivity and child competency when treating children.

The current study indicates that the DFA sensitivity drops with increasing age, with DFA specificity rising accordingly, i.e., fewer patients are correctly defined as anxious. Simultaneously, more patients are correctly defined as non-anxious. The finding is congruent with the dental fear prevalence, which normally decreases with age (Cianetti et al. 2017).

Preventing DFA often requires a high level of skill and competency to balance what leads to prevention, especially where young children are concerned. It is often considered a simple task for the dental staff to strive to prevent DFA for each dental setting. However, the methods for the prevention of DFA may be unintentionally misused by the general dental practitioners due to habitual and reoccurring daily praxis. There is substantial knowledge on how to prevent DFA, but this knowledge may not be consistently applied by dental practitioners.

A limitation of this study is that comparable data are lacking to match the methodology, a prospective and prolonged study design. The advantage of this study is that each age cohort holds a substantial number of young patients, facilitating a careful generalization of the findings.

The current findings generate further scientific questions for future research. Readily applicable DFA tools are needed to be systematically evaluated in clinical settings.

## Conclusion

Considering the limitations of the present study, the following conclusions can be made:

- Consistent DFA tools are needed to be used in clinical and research settings.
- Children should be addressed regarding their DFA experience in every dental treatment session.
- A knowledge gap exists regarding the congruence of self-reports and proxy reports in longitudinal, prospective studies.
- The dental professionals’ sensitivity to the young patient’s fear is crucial for a successful treatment outcome.
